# Severe Late-Onset Neutropenia in a Patient With Multiple Sclerosis Treated With Ocrelizumab

**DOI:** 10.7759/cureus.99574

**Published:** 2025-12-18

**Authors:** Mohamed K Mansour, Raju Raghunathan, Ali Mohamed, Hatem Ahmed, Yousif Hassan

**Affiliations:** 1 Internal Medicine, Rochester Regional Health, Rochester, USA; 2 Hospital Medicine, Cleveland Clinic, Abu Dhabi, ARE; 3 Internal Medicine, Royal Hobart Hospital, Hobart, AUS; 4 Internal Medicine, Tower Health Medical Group, Phoenixville, USA

**Keywords:** late onset neutropenia, ms (multiple sclerosis), multiple sclerosis treatment, ocrelizumab, severe neutropenia

## Abstract

Ocrelizumab, a humanised monoclonal antibody targeting cluster of differentiation 20 (CD20), is widely used to treat multiple sclerosis (MS). While it is effective in reducing relapse rates, adverse effects such as late-onset neutropenia can pose significant clinical challenges. We report the case of a 36-year-old female with a history of relapsing-remitting MS (RRMS) who developed severe neutropenia following ocrelizumab administration. The patient presented with fever, fatigue, and oral aphthous ulcers, with laboratory tests confirming an absolute neutrophil count (ANC) of 0.11 × 10⁹/L. Prompt initiation of broad-spectrum antibiotics and granulocyte colony-stimulating factor (G-CSF) resulted in significant clinical improvement. This case highlights the importance of monitoring blood counts in patients receiving ocrelizumab and the need for timely intervention for late-onset neutropenia (LON).

## Introduction

Multiple sclerosis (MS) is a chronic immune-mediated disorder characterized by demyelination and neurodegeneration within the CNS, leading to sensory, motor, and cognitive dysfunction [1,2]. Among disease-modifying therapies, ocrelizumab, a recombinant humanised monoclonal antibody targeting cluster of differentiation 20 (CD20)-positive B cells, has demonstrated significant efficacy in reducing relapse rates and slowing disability progression in both relapsing-remitting and primary progressive MS [3,4].

While generally well tolerated, ocrelizumab has been associated with several adverse effects, including infusion reactions, infections, and rare hematologic complications [5,6]. One emerging concern is late-onset neutropenia (LON), a condition characterized by a decrease in absolute neutrophil count (ANC) to <1.5 × 10⁹/L occurring more than four weeks after the last dose of therapy [7,8]. Although the mechanism remains unclear, proposed explanations include immune-mediated marrow suppression and altered cytokine signaling leading to transient myelosuppression [9]. Recognizing and managing this complication is crucial, as neutropenia may predispose patients to severe infections.

This report presents a case of LON following ocrelizumab therapy in a patient with relapsing-remitting MS (RRMS) and discusses its clinical significance in light of current literature.

## Case presentation

A 36-year-old female with a history of RRMS, diagnosed in 2018, had been receiving ocrelizumab infusions since 2021. She had no other comorbidities and was not on any other medications. She presented to our hospital on July 11, 2024, with a five-day history of fever (38°C), fatigue, and sore throat. On physical examination, she was found to have oral aphthous ulcers and enlarged tonsils with purulent exudate. The patient had received her last dose of ocrelizumab (600 mg IV) on May 10, 2024, and her blood counts at that time were within normal limits.

Laboratory results on admission, as outlined in Table 1, revealed a significant drop in her WBC count (WBC: 1.7 × 10⁹/L) and ANC (0.11 × 10⁹/L), indicating severe neutropenia. Her hemoglobin was 95 g/L, and her platelet count was 395 × 10⁹/L. Inflammatory markers were markedly elevated, with a CRP level of 236 mg/L and a procalcitonin level of 2.5 mcg/L. Peripheral blood smear confirmed the neutropenia without any atypical or immature cells. A CT scan of the neck ruled out peritonsillar abscess. After a thorough review of her medical records, it was confirmed that she had not received any medications known to cause severe neutropenia. The most plausible explanation for her neutropenia was the ocrelizumab infusion she received approximately two months prior.

**Table 1:** Laboratory investigations during hospital course. Note the recovery of the absolute neutrophil count (ANC) after therapy with granulocyte colony-stimulating factor (G-CSF).

Hospital day	Test	Result	Reference range
Admission (Day 1)	WBC	1.7 × 10⁹/L	4.0-11.0 × 10⁹/L
	ANC	0.11 × 10⁹/L	1.5-8.0 × 10⁹/L
	Hemoglobin	95 g/L	120-160 g/L
	Platelets	395 × 10⁹/L	150-450 × 10⁹/L
	CRP	236 mg/L	<10 mg/L
	Procalcitonin	2.5 mcg/L	<0.1 mcg/L
Post-G-CSF (Day 2)	WBC	12.2 × 10⁹/L	4.0-11.0 × 10⁹/L
	ANC	1.26 × 10⁹/L	1.5-8.0 × 10⁹/L
One-month follow-up	WBC	4.2 × 10⁹/L	4.0-11.0 × 10⁹/L
	ANC	2.54 × 10⁹/L	1.5-8.0 × 10⁹/L

The patient was immediately started on intravenous piperacillin-tazobactam (4.5 g every 8 hours) and intravenous acyclovir (10 mg/kg every 8 hours) to cover for bacterial and viral infections. In addition, she was given subcutaneous G-CSF 300 mcg daily to address the neutropenia. An extensive infectious workup, including chest X-ray, blood and urine cultures, bacterial and viral throat cultures, fungal cultures, and tests for serum beta-D-glucan, galactomannan, and cytomegalovirus (CMV) PCR, was negative for any infectious agents. Serum folate and vitamin B12 levels were within the normal range. Bone marrow biopsy and flow cytometry revealed no evidence of hematologic malignancy.

Within 24 hours of receiving G-CSF, her WBC improved to 12.2 × 10⁹/L, and her ANC rose to 1.26 × 10⁹/L, as shown in Table 1. Clinically, her fever subsided, and she experienced resolution of her oral ulcers and throat discomfort. The patient completed five days of intravenous antibiotic therapy with piperacillin-tazobactam and acyclovir before being discharged in good condition on oral valacyclovir, to complete a total 10-day course of therapy.

The patient remained healthy at her follow-up visit one month later in the neurology clinic. Her complete blood count at that time, as outlined in Table 1, showed a WBC of 4.2 × 10⁹/L and an ANC of 2.54 × 10⁹/L. Given the episode of severe neutropenia associated with ocrelizumab, in consultation with the patient, it was decided to switch her multiple sclerosis therapy to cladribine.

## Discussion

Ocrelizumab is a recombinant humanised monoclonal antibody that selectively targets CD20, a glycosylated phosphoprotein expressed on the surface of B lymphocytes but not on plasma cells or neutrophils [1]. This mechanism closely resembles that of rituximab, a well-established treatment in rheumatic diseases [1]. Approved by the FDA in March 2017 for the management of relapsing and primary progressive MS, ocrelizumab is administered intravenously every six months [1]. Despite its therapeutic promise, the precise mechanisms underlying its efficacy remain incompletely understood. Current hypotheses suggest that ocrelizumab may mediate its effects through antibody-dependent cellular cytotoxicity and complement-mediated lysis of CD20-expressing cells [2].

Notably, adverse effects such as infusion reactions, infections, and malignancies have been documented with ocrelizumab use [1]. While several studies have associated rituximab with late-onset neutropenia, data on ocrelizumab’s role in this condition are limited [1-7]. Late-onset neutropenia is characterised by an ANC of less than 1.5 × 10⁹/L, developing more than four weeks after the last administration of the drug, in the absence of other identifiable causes, and following a previously normal ANC [1,2]. In a clinical trial for primary progressive MS, neutropenia occurred in 13% of patients receiving ocrelizumab, compared to 10% in the placebo group [5].

The pathophysiology of late-onset neutropenia associated with ocrelizumab therapy remains unclear [6-10]. Some studies indicate that it may result from maturation arrest in the myeloid lineage and alterations in growth factor dynamics, leading to increased production of B cell-activating factors. This shift may redirect haematopoiesis in the bone marrow toward B-cell production at the expense of granulocyte formation, consequently reducing neutrophil counts [3,6,7]. Notably, the onset of late-onset neutropenia following ocrelizumab administration can range from 2.5 to 10 months [7]. Current literature suggests that the administration of G-CSF (filgrastim) for managing late-onset neutropenia offers minimal benefit in altering its overall course, although it may accelerate its resolution [2].

The first reported case of late-onset neutropenia related to ocrelizumab involved a 35-year-old female with relapsing MS who presented with neutropenic fever and mucositis 73 days post-therapy, exhibiting an ANC of 0 × 10⁹/L. Treatment with cefepime, acyclovir, and a single subcutaneous dose of filgrastim resulted in ANC improvement, allowing for discharge two days post-admission [1]. Similarly, Zanetta C et al. documented a 26-year-old treatment-naïve female with relapsing MS who developed neutropenic fever, mucositis, and headache three months after her last ocrelizumab cycle, presenting with an ANC of 0 × 10⁹/L. Her condition improved within two days of initiating broad-spectrum antibiotics, with normal blood counts documented 14 days after hospital discharge [2].

Furthermore, Baird-Gunning J et al. reported a case of a 34-year-old male with primary progressive MS who experienced neutropenic enterocolitis 42 days after receiving ocrelizumab, with an ANC of 0.5 × 10⁹/L. The patient was treated with intravenous broad-spectrum antibiotics and G-CSF, leading to normalisation of ANC within five days [6]. In another case by Rauniyar R et al., a 38-year-old male with primary progressive MS developed severe late-onset neutropenia three months after his last ocrelizumab dose, presenting with an ANC of 0 × 10⁹/L. He was managed with empiric intravenous broad-spectrum antibiotics and filgrastim, which raised his ANC to 2.7 × 10⁹/L. Notably, his ANC dropped again to 0.2 × 10⁹/L without further ocrelizumab treatment, prompting additional filgrastim therapy that restored his ANC to 2.1 × 10⁹/L [7].

Numerous aetiologies for neutropenia were excluded in our patient, including infectious causes (viral, bacterial, and fungal infections), nutritional causes (vitamin B12 and folate deficiency), and myelodysplasia. This leaves ocrelizumab as the likely aetiology for her neutropenia, particularly in the absence of a history of other medication coadministration that could potentially contribute to neutropenia. Late-onset neutropenia associated with ocrelizumab therapy is often self-limited and can vary in severity. However, the literature advocates using empirical broad-spectrum antibiotics and G-CSF to enhance recovery and improve outcomes [6,7]. Though concerns about potential exacerbations of MS have been raised regarding the use of granulocyte colony-stimulating factor, the overall benefits appear to outweigh the risks, as evidenced by multiple positive treatment responses in the reported cases [7,8].

## Conclusions

In summary, late-onset neutropenia is a significant complication associated with ocrelizumab therapy, necessitating close monitoring and prompt management. This case underscores the importance of recognising neutropenia in patients receiving ocrelizumab, as early intervention with G-CSF and appropriate antibiotic therapy can lead to favourable clinical outcomes. Clinicians should remain vigilant for haematological adverse effects and consider alternative therapies for patients experiencing severe neutropenia in the context of ocrelizumab treatment. Further studies are warranted to elucidate the underlying mechanisms of this complication and establish clear management protocols to optimise patient care in this population.
